# Relationship between economic liberalization and intellectual property protection with regional innovation in China. A case study of Chinese provinces

**DOI:** 10.1371/journal.pone.0259170

**Published:** 2022-01-27

**Authors:** Muhammad Qayyum, Yuyuan Yu, Tingting Tu, Mir Muhammad Nizamani, Afaq Ahmad, Minhaj Ali

**Affiliations:** 1 School of Economics, Hainan University, Haikou, China; 2 School of International Economics and Trade, Central University of Finance and Economics, Beijing, China; 3 School of Business Administration, Zhongnan University of Economics and Law, Wuhan, China; 4 School of Life and Pharmaceutical Sciences, Hainan University, Haikou, China; 5 Institute of Business, Management and Administrative Sciences, The Islamia University of Bahawalpur, Bahawalpur, Pakistan; 6 School of Economics, Zhongnan University of Economics and Law, Wuhan, China; Szechenyi Istvan University: Szechenyi Istvan Egyetem, HUNGARY

## Abstract

International openness can affect regional innovation through more export opportunities, enhanced import competition and the spillover effects of foreign direct investment. Many studies have been conducted based on different countries for capturing the determinants of regional innovation, but very little literature is available with contradictory findings for the case of China. Based on 19 years’ panel data of 31 Chinese provinces, this paper analyzes the impact of international openness on regional innovation measured by the number of patent grants. The positive effects of overall trade and a higher proportion of exports and imports to GDP are significant and robust across different model specifications, indicating that an increase in international openness can promote regional innovating activities in China. The causal relationship of all the variables depicted by path analysis matches the results of the system GMM model. Higher intellectual property protection provides each region with the opportunity to obtain economic benefits from innovation and then make a higher investment in R&D activities. Besides, the lag effect of regional innovation capability can also explain a large part of local innovating activities. In our subsample regressions, the positive effect of trade openness on innovation is majorly manifested in developed areas like eastern provinces.

## 1. Introduction

Intellectual property is protected under the legislation, such as patents, copyrights and trademarks, which allow individuals to gain recognition or to benefit financially from what they invent or create [1]. Over the past few years, the number of Chinese patent applications to WIPO (World Intellectual Property Organization) has risen sharply in recent years and China has ranked among the top 10 countries in filing international patents. Chinese patents filed to the WIPO accounted for nearly 43% of the world’s total patent application in 2017, far higher than the United States (19%), Japan (10%) and South Korea (7%) (WIPO database, 2017). Apart from the international patent application, the number of domestic patents filed to China’s Intellectual Property Office (SIPO) has also soared dramatically, including invention patents, utility model patents, trademarks and designs. Many researchers have used the number of patent applications and grants as a measure of productivity and innovation ability of a country [2,3]. However, the relationship of regional innovation with international openness and intellectual property protection is poorly studied in the current literature for China.

Many pieces of literature have identified several key channels through which international openness can exert its influence on innovation. Different from previous studies, we consider three aspects of international openness separately while examining its effects, including the larger proportions of total trade, export and import. Export opportunities can promote innovation through two channels: the “market-size” effect and the “learning by exporting” effect [4]. Driven by the “market size” effect, firms can obtain higher economic rents after they have more access to the international market. Shu and Steinwender [5] summarized that import competition could lead to two opposite effects on innovation: “Schumpeter effect” (the inclination to decrease innovating activities because import competition reduces economic rents) and “escape-competition effect” (the inclination to increase innovation for higher pre-competition rents). The overall effects of import competition depend on the relative power of these two opposite effects. In addition, Aitken and Harrison [6] pointed out that there are two opposite IFDI effects: the “own-plant” effect (increase in foreign equity participation can promote the productivity of self-owned plants) and the “market-stealing” effect (increase in IFDI has significantly negative impact on the productivity of domestic firms). The international openness effects on innovation are closely related to local IPR protection and IPR protection plays an important role in moderating the relationship between international openness and firm-level innovation [7].

We establish an econometric model based on Chinese provincial-level panel data from the China Statistical Yearbook. In the econometric specifications, the number of patent grants is explained by variables of interest and other variables, including GDP, local R&D investment and the number of local college graduates. In the beginning, we use the Ordinary Least Square (OLS) method and Fixed-effect (FE) model to estimate the coefficients of each variable. The results show that overall trade openness, export and import have a significantly positive impact on the number of invention patent grants. However, such econometric specifications may suffer from severe endogeneity problems, given the fact that international openness and innovation are essential determinants of each other [8,9].

Considering the econometric techniques developed by Yu and Qayyum [9], Yang et. al. [10], Qayyum et. al. [11], we introduce the lagged one-period variable of the dependent variable into the original model and estimate this dynamic panel model with the system GMM one-step method. Standard dynamic panel models require the inclusion of lagged dependent variable. So we introduce the lagged one-period dependent variable (L.innovation) in the model as shown in Eqs 3 and 4. Consequently, Eqs 3 and 4 now suffer from severe endogeneity problem due to the inclusion of lagged dependent variable. To solve this problem, GMM approach allows us to avoid the endogeneity problem by using proper instrumental variables. One important step for the system GMM method is to identify the endogenous variable and exogenous instrumental variable. Here, we select the lagged two-period variable of international openness as the instrumental variable for further estimation. In this paper, we consider the lagged two-period variable of international openness as the instrumental variable in GMM estimation. After considering the potential endogeneity problem, the empirical results of overall trade openness, export and import are still consistent with the previous OLS method and FE model. However, the estimated coefficient of IFDI is not robust under different model specifications. This conclusion about the minor IFDI effect matches the empirical findings of Su and Liu [12], Farla et. al. [13] and Li et. al. [14].

In the GMM model, IPR variables have positive and significant coefficients and this indicates that higher IPR protection grants local firms the opportunity to obtain the economic rents of innovation and then make a higher investment in R&D activities. We have also included path analysis to capture the causal relationship of all variables. We also test the impact of international openness on innovation for three Chinese regions and the results show that the positive effect of export and import on innovation are majorly manifested in developed areas like eastern provinces. It has been widely recognized that international openness plays an important role in innovating activities and there is a significant interaction between the development of international openness and regional innovation. However, attempts to test the impact of international openness and IPR protection on innovation by considering specific provincial-level conditions empirically are rare. This can be an important research gap because regional differences are considerably huge in economic development, IPR protection, R&D investments and research personnel.

The goal of this research paper is to explore the impact of international openness and Intellectual Property Rights (IPRs) protection on regional innovation based on a provincial panel dataset. The proposed study questions incorporated in this research are: (1) What are the determinants of such fast progress in patent applications and grants in China? (2) Is it that China’s innovation is simply driven by importing technology instead of developing its own innovating ability?

The rest of this paper is organized as follows: Section 2 reviews previous studies related to the impact of international openness and IPR protection on innovation; section 3 describes the data source and model specification; section 4 presents the empirical findings and analysis; section 5 concludes.

## 2. Literature review

The impacts of trade openness on innovation can be summarized as import competition and export opportunities [5]. Most empirical studies find a positive relationship between export opportunity and innovation. Shu and Steinwender [5] pointed out that export opportunities can promote innovation through two channels: the “market-size” effect and the “learning by exporting” effect. Driven by the “market size” effect, firms can obtain higher economic rents after they have more access to the international market. Geng and Kali [15] identified the “market size” effect of export opportunity on innovation and productivity in Canadian plants. Specifically, the elimination of the US tariff under the terms of the Canada-US Free Trade Agreement (FTA) has significantly increased the investment of Canadian firms in product innovation due to the reduction of export costs. By using a panel data of Spanish manufacturing firms between 1990 and1999, Freixanet et.al. [16] found there is a complementary relationship between export and innovation because export activities can enhance innovation via “learning by doing” effect and innovation activities can help firms to produce more high-quality products and to enter more marketplaces. On the one hand, import competition from low-income countries may hinder the innovation of high-income countries because cheaper imports could reduce potential economic rents arising from product innovation or process innovation of domestic firms, which is also known as the “Schumpeter effect” [5]. Based on a matched dataset of firm-level US utility patent grants, trade and production, Dorn et. al. [17] concluded that import competition from China has a significantly negative impact on the patenting activities of the US trade-exposed firms. Meanwhile, Dorn et. al. [17] found that those firms have also experienced a significant decline in employment, R&D investment, sales revenue and employment. Kueng et al. [18] also made a similar conclusion by using the micro-level data of Canadian manufacturing firms between 1999 and 2005. Based on Chinese firm-level data from 1998 to 2007, Liu and Qiu [19] took China’s accession to the WTO in 2001 as a quasi-natural experiment of dramatic import tariff cut and found out that trade shocks in terms of imported intermediate input market led to fewer innovation activities within Chinese firms. They believed that cheaper intermediate input imports with high quality can be gross substitutes of firm innovation because Chinese firms would choose to buy the technology contained in the products instead of inventing on their own. On the other hand, increased import competition may motivate firms to innovate more via improvement in corporate management performance or strategic redeployment, which has been addressed as the “escape-competition effect” by Shu and Steinwender [5]. Henn et al. [20] have shown in their models with a five-year interval between 1990 and 2005 that trade openness measured by lower import tariff can significantly encourage product quality upgrading of the United States. Kueng and Yang [18] attributed quality upgrading and innovation to the market segmentation in product space and geographical location. Faced with import competition, firms in rich economies are inclined to specialize in a specific segmented market and produce highly differentiated products, which can reflect a dynamic productivity gain. In addition, Im et al. [21] found that there is an inverted U-shaped relationship between competition, measured by Lerner Index or price-cost margin, and innovation by using UK panel data. More specifically, Im et al. [21] pointed out that there is a higher possibility that the escape-competition effect dominates over the Schumpeter effect under low competition level while the Schumpeter effect becomes more dominant with the increase in competition level. By using a larger data of US publicly listed manufacturing firms based on the theoretical model of Im et al. [21], the empirical results of Howell [22] show that the negative impact of competition is rather mild on innovation. This finding can be reconciled with the findings of Im et al. [21] by making some modifications that UK firms are more technologically neck-and-neck than their US counterparts. However, their conclusion has been challenged by Tammi et al. [23] because they pointed out that the findings of Im et al. [21] are sensitive to the selection of proxies while the inverted U-shaped relationship can be better explained by the Herfindahl index but not by the price-cost margin.

Another critical aspect of international openness is the elimination of domestic barriers to inward foreign direct investment (IFDI). As for the relationship between IFDI and innovation, there are no unified conclusions about the impact of IFDI on innovation. The technological content embedded in the products from rich countries will flow to developing countries and developing countries can obtain technology through reverse engineering, which has been widely regarded as the technological spillover effect. Dong et al. [24] found that there is a positive spillover effect of IFDI on China’s provincial-level innovation, which is measured by the number of domestic patent applications. This indicates that China has been developing its own ability on innovation instead of merely importing foreign technology. Antonietti et al. [25] also made the same conclusion by using Italian provincial level and industrial level greenfield IFDI data and they concluded that the positive relationship between greenfield IFDI and local patenting only exists in the service sector. However, some empirical results show that there is no significant impact of IFDI on innovation. Using panel data on Venezuelan plants, Aitken and Harrison [6] found that the net effect of IFDI was quite small after summing up the “own-plant” effect (increase in foreign equity participation can promote the productivity of self-owned plants) and the “market-stealing” effect (increase in IFDI has a significantly negative impact on the productivity of domestic firms). Pradeep et al. [26] pointed out that positive spillover effects of IFDI did not present robust results and the impact of horizontal IFDI on technology transfer is insignificant based on the data of Indian manufacturing firms.

As there is a large technological gap between developed and developing countries, trade and FDI have become an important channel for developing countries to acquire advanced technology. At the same time, intellectual property right (IPR) protection has been proved to have a significant impact on innovation because a relatively mature intellectual property protection regime often leads to higher trade volume and FDI to a certain extent, thus promoting the global flow of technology [27,28]. Aghion et al. [29] pointed out that for those exporting companies that adopt a price discrimination strategy, the importing countries’ enhancement of IPR protection brings them stronger market power and larger market scale, which are known as the “market-power” effect and “market-expansion” effect. Because both effects are possible to occur, the effect of IPR protection on trade is uncertain. Fatma [30], argued that countries with weak imitation ability and strong intellectual property protection also have lower imitation threats so that the market power effect dominates. This means that trade volume will be reduced when IPR protection is enhanced. On the contrary, countries with strong imitation ability and weak IPR protection have a higher imitation threat, so the market expansion effect dominates and trade volume will be increased when intellectual property protection is enhanced. The empirical results of Li et al., [31] show that the enhancement of IPR protection in importing countries improves the extensive margin of China’s exports, but has a negative impact on the intensive margin of China’s product exports. The impact of intellectual property protection on the exports of technology-intensive industries is mainly reflected in the extensive margin. Sun et al., [32] used the Propensity Score Matching method (PSM) and Difference-In-Difference method (DID) to calculate the impact of intellectual property protection reform in Argentina, Brazil, Philippines and other countries on trade volume. The results of the gravity model show that IPR protection reform in developing countries can significantly increase the total imports of developing countries from developed countries by 1.1% to 2.7%. The empirical results of Zhao et al., [33] show that institutional factors like IPR protection and privatization have a significantly positive impact on Chinese firms’ patenting activities while IPR protection plays an important role in moderating the relationship between privatization and firm-level innovation. Theoretically, Anser et al., [34] constructed a dynamic endogenous growth model with a single final good and concluded that there is an inverted U-shaped relationship between IPR protection and innovation. Teece [35], further explained that IPR protection grants firm the opportunity to obtain high economic rents from innovation, while this also indicates greater difficulty in future innovation because of limited technological spillover.

## 3. Data and methodology

### 3.1. Data source and descriptive analysis

This paper undertakes a comprehensive study of the impact of international openness and IPR on regional innovation of 31 Chinese provinces. Based on the previous study, we employ the ratio of trade, import and export to GDP as the proxies of international openness and the number of invention patent grants as the proxies of regional innovation. Our empirical results are based on provincial-level panel data covering 31 Chinese provinces from 1998 to 2016. China Statistical Yearbook provides data on the number of patent grants for each province as well as the data on trade, import, export, IFDI, GDP, number of college graduates and R&D investments. As GDP data is denominated in Chinese currency and international trade data in US currency, we adjust GDP by using the annual CNY-USD exchange rate, which is extracted from China Statistical Yearbook. Following the practice of Lin et al., [36], we use the percentage of technological property rights transactions to local GDP protection as the proxy for local IPR protection. China Statistical Yearbook provides data on regional technological property rights transactions.

Definition of key variables is provided in Table 1. Table 2 illustrates the descriptive statistics of key variables, including the natural logarithm form of the number of invention patent grants (*INV*), the ratio of total trade (*Rtrade*), import(*Rimport*) and export (Rexport) to GDP, IFDI flow (*ifdi*), R&D investment (*RD*), GDP (*gdp*) and the number of college graduates (*graduates*). Table 3 shows the gap between three major regions in China. It can be seen that eastern provinces have the highest values in most variables among the three regions, while the western provinces have the lowest values in most values.

**Table 1:** Definition of key variables.

Variable Name	Explanation
Dependent variable INV	log (the number of grants of the invention patents)
Independent variable Rtrade	Ratio of total trade to GDP = Total trade / GDP, %
Rexport	Ratio of exports to GDP = Exports / GDP, %
Rimport	Ratio of imports to GDP = Exports / GDP, %
RIPR	Ratio of technological property rights transaction to GDP, %
GDP	Gross Domestic Product of each province in billion USD
RD	Local R&D expenditure in billion USD
Graduates	Number of college graduates in 10,000
IFDI	Inward Foreign Direct Investment flow in billion USD

**Table 2:** Descriptive statistics of key variables.

variable	mean	Quantile 5%	Quantile 50%	Quantile 95%	sd	min	max	N
INV	6.087	2.773	6.040	9.337	2.005	0	10.62	589
Rtrade	30.70	4.778	12.72	126.2	39.05	3.205	206.7	589
Rexport	15.85	2.721	7.063	58.86	18.64	1.484	94.17	589
Rimport	14.85	1.373	5.415	68.37	23.41	0.388	175.4	589
RIPR	0.881	0.051	0.368	2.840	1.802	0	15.35	589
gdp	160.8	6.565	83.62	526.1	200	1.105	1217	589
ifdi	71.06	1.144	24.10	324.3	125.2	0.256	879.9	589
RD	2.697	0.021	0.773	12.35	4.845	0.001	30.64	589
graduates	12.89	0.580	9.500	39.09	12.01	0.080	50.91	589

**Table 3:** Mean values of key variables in different regions.

variable	Eastern provinces	Central provinces	Western provinces	National wide
Provinces	Beijing, Fujian, Guangdong, Hainan, Heibei, Jiangsu, Liaoning, Shandong, Shanghai, Tianjin, Zhejiang	Anhui, Heilongjiang, Henan, Hubei, Hunan, Jiangxi, Jilin, Shanxi	Chongqing, Gansu, Guangxi, Guizhou, Neimenggu, Ningxia, Qinghai, Shaanxi, Sichuan, Tibet, Xinjiang, Yunan	
INV	7.021	6.298	5.088	6.087
Rtrade	62.273	10.471	10.667	30.70
Rexport	33.064	5.821	6.761	15.85
Rimport	34.209	4.650	3.906	14.85
RIPR	1.506	0.439	0.727	0.881
gdp	256.546	150.781	79.694	160.8
ifdi	165.736	26.837	13.742	71.06
RD	5.277	1.845	0.900	2.697
graduates	16.277	16.582	7.331	12.89

### 3.2. Econometric model

This paper constructs a baseline econometric model to examine the impact of international openness on regional innovation as follows:

$$innovation_{jt}=\beta_{0}+\beta_{1}IO_{jt}+\beta_{2}IPR_{jt}+\beta_{3}X_{jt}+\varepsilon_{jt}$$ (1)

$$innovation_{jt}=\beta_{0}+\beta_{1}IO_{jt}+\beta_{2}IPR_{jt}+\beta_{3}X_{jt}+\beta_{4}IO_{jt}*IPR_{jt}+\varepsilon_{jt}$$ (2)

Eqs (1) and (2) test the impacts of international openness and IPR as well as their interaction term on regional innovation by using panel data of 19 years covering 31 Chinese provinces. Subscript *j* represents the province while *t* represents the year. The dependent variable innovation is measured by the number of patent grants of each province in the natural logarithm form to reduce the influence of outliers and specification errors. Variable IO represents the extent of international openness of each province, which is measured by the natural logarithm form of the percentage of total trade (*Rtrade*, *%*), import (*Rimports*, *%*) and export (*Rexports*, *%*) to local GDP. IPR is the percentage of local technological property rights transaction to GDP (%). Vector X includes local GDP (*gdp*), R&D expenditure (*RD*), IFDI flow (*ifdi*) the number of college graduates (*graduates*). Therefore, both of Eqs (1) and (2) are under the log-linear specification.

On the one hand, the panel data employed in this study has relatively large cross-sectional variation and OLS estimation can be subject to unobservable heterogeneity bias [37,38]. Fixed effects model can control time-invariants and region-invariants so that unobservable heterogeneity bias can be partly reduced [39,40]. On the other hand, many studies have found that productivity improvement brought by technological advancement and innovation has a significant trade effect. Regional engagement in international economic activities and innovation activities are often interrelated with each other [41–43]. Using the data of 2822 Turkish manufacturing firms and the multinomial logit estimation method, Lo Segarra-Blasco et al., [44] found that product innovation contributes more to firms’ export performance than process innovation and firms with proper innovation strategy have a higher export probability to high-income countries. Therefore, OLS estimation and fixed effects regression in Eqs (1) and (2) may suffer from severe endogeneity problems. To overcome potential endogeneity problems, we add the lagged value of innovation into Eqs (1) and (2) to dynamically study the relationship between international openness and regional innovation.


$$innovation_{jt}=\beta_{0}+\delta_{1}L.innovation_{jt}+\beta_{1}IO_{jt}+\beta_{2}IPR_{jt}+\beta_{3}X_{jt}+\varepsilon_{jt}$$ (3)

$$innovation_{jt}=\beta_{0}+\delta_{1}L.innovation_{jt}+\beta_{1}IO_{jt}+\beta_{2}IPR_{j}+\beta_{3}X_{jt}+\beta_{4}IO_{jt}*IPR_{jt}+\varepsilon_{jt}$$ (4)

*L*.*innovation* represents the lagged one-period of the explained variable. With the inclusion of lagged explained variables, Eqs (3) and (4) become dynamic panel-data models and traditional estimation methods like OLS would yield inconsistent and biased estimates because the lagged variable is often correlated to the error terms. The Generalized Method of Moments (GMM method) proposed by Wang et al., [45] has been useful in solving the above estimation error. GMM method can be divided into one-step estimation and two-step estimation according to different weight matrices. Radivojević et al., [46] believe that the standard error of two-step GMM estimates has significant downward biases in the case of limited samples. Besides, for a dynamic panel data model, both the difference GMM and system GMM approaches have been used while Rezgallah et al., [47] pointed out that the system GMM method can overcome a large part of the disappointing outcomes because the lagged variable is not an ideal instrument in the first-order difference GMM model. Therefore, we use the system GMM method with one step to estimate Eq (2). A critical step in employing system GMM in mode estimation is to determine endogenous variables and predetermined variables. Based on the research aim of this paper, we select the proxies of international openness as endogenous variables and other variables as predetermined variables.

Path analysis is a type of multiple statistical analysis of regression used by examining the relationship between the dependent variable and two or more independent variables to evaluate the causal model. The size and importance of the causal relationship between the variables can be estimated through the use of this technique. Researchers can better comprehend the causal relationship between various variables through path analysis. First, to visually represent the relationship between variables, researchers should draw a chart. In theory, path analysis is useful because it forces us to specify the relationship between all of the measured independent variables, unlike other techniques. This contributes to a model that can demonstrate a causal mechanism, in which the independent variables have a direct and indirect effect on the dependent variables. Path analysis was established by the geneticist Sewall Wright in 1918. Over time, other physical sciences and social sciences including sociology have adopted this technique. Path analysis is also known as the causal model, covariance structure analysis, and latent variable model Loehlin, [48]. We use R software following packages lavaan, semPlot, OpenMx, tidyverse, knitr, kableExtra, and GGally for conducting path analysis in this paper.

## 4. Results and discussion

### 4.1. Baseline results

We have used a combination of four advanced econometric models for statistical estimation, which are named as: OLS, fixed effect (FE), System GMM (SGMM), and path analysis. Table 4 contains the estimation results of Eqs (1) to (4) for the impacts of overall trade and IPR.

**Table 4:** Baseline results (*Rtrade)*.

	(1)	(2)	(3)	(4)	(5)	(6)
	OLS	FE	SGMM	OLS	FE	SGMM
L.INV			0.844***			0.887***
			(23.07)			(30.45)
Rtrade	0.00888***	0.0115***	0.0015**	0.0103***	0.0145***	0.00187**
	(3.88)	(4.01)	(2.83)	(3.66)	(4.07)	(3.25)
RIPR	0.296***	0.297***	0.0383**	0.331***	0.359***	0.0447***
	(6.91)	(6.59)	(3.29)	(5.58)	(5.78)	(4.81)
ifdi	0.00243**	0.00309***	0.000255	0.00225**	0.00284**	0.0000936
	(2.93)	(3.55)	(0.85)	(2.62)	(3.20)	(0.42)
gdp	0.00544***	0.00593***	0.000335	0.00541***	0.00591***	0.000220
	(6.35)	(6.76)	(1.45)	(6.30)	(6.75)	(1.12)
RD	-0.144***	-0.159***	-0.0112	-0.138***	-0.150***	-0.00827
	(-4.76)	(-5.21)	(-1.56)	(-4.46)	(-4.82)	(-1.27)
graduates	0.0803***	0.0748***	0.0147***	0.0798***	0.0731***	0.0111***
	(9.84)	(8.63)	(3.97)	(9.76)	(8.37)	(4.32)
Rtrade_RIPR				-0.000432	-0.000751	-0.000195
				(-0.86)	(-1.44)	(-1.80)
Sargan test			119.72***			157.39***
AR(1)			0.028			0.029
AR(2)			0.110			0.103
_cons	3.857***	3.766***	0.879***	3.818***	3.681***	0.695***
	(25.84)	(37.38)	(4.97)	(24.39)	(31.58)	(4.47)
N	589	589	558	589	589	558

Note: This table reports the estimation results of the impact of overall trade openness. We used the OLS method and FE model to estimate Eqs (1) and (2) while we use the system GMM one-step method to estimate Eqs (3) and (4). For the system GMM method, we employ the lagged two-period international openness proxies as endogenous variables and other variables as predetermined variables. We report the chi2 statistics for the Sargan test of over-identification and the P values for AR (1) and AR (2) test. It is assumed that the error term in the system GMM model has significant first-order auto-correlation and insignificant second-order auto-autocorrelation. T statistics are reported in parentheses.

*, **, and *** indicates statistical significance at the 5%, 1%, and 0.1% level, respectively.

As can be seen from Table 4, with other factors being controlled, both *Rtrade* and *RIPR* have significant and positive impacts on the number of invention patent grants, which are also robust across different estimation methods and model specifications. In the system GMM model in column (3), the magnitudes of the effect of trade openness become lower: a 1-unit increase in trade openness and IPR protection can result in a 0.15% and 3.83% increase in the number of invention patent grants. This is because 84.4% of the fluctuation in the number of invention patent grants can be explained by the lag effect of regional innovation capacity. Positive and significant coefficients of IPR mean higher IPR protection grants local firms the opportunity to obtain the economic rents of innovation and then make a higher investment in R&D activities. Besides, it can be seen from the results in columns (4) to (6) that the impact of the interaction term of *Rtrade* and *RIPR* is insignificant, which means that there is no sufficient evidence to prove the existence of a mediating effect between international openness and IPR protection. As for the impact of IFDI, OLS estimation and FE model show that the coefficients of *ifdi* variable are positive and significant in statistics, indicating positive spillover effect, or own-plant effect, dominates over positive spillover effect like the market stealing effect. However, in columns (3) and (6), the coefficient of *ifdi* becomes insignificant in the system GMM model, which means that the impact of *ifdi* is not robust and varies with model selection. This result is also consistent with many studies that hold IFDI effect becomes very small when the positive and negative spillover effect is summed up so that there is no evidence of the significant impact of IFDI on innovation [49–53].

To dissect the effects of international openness, we also tested the importance of export and import to regional innovation. As shown in Tables 5 and 6, both *Rexport* variable and *Rimport* variable show significantly positive coefficients in OLS estimation and FE model. With the endogeneity problem being considered, the coefficients of *Rexport* variable and *Rimport* variable are still consistent, showing strong robustness in terms of model specification. The empirical results of the *Rimport* variable imply that the Schumpeter effect dominates over the escape-competition effect in Chinese provincial-level panel data. Higher import competition can promote regional innovating activities because Chinese provinces are more motivated to escape the economic rent paid for import competition.

**Table 5:** Baseline results (*Rexport)*.

	(1)	(2)	(3)	(4)	(5)	(6)
	OLS	FE	SGMM	OLS	FE	SGMM
L.INV1			0.834***			0.907***
			(29.36)			(33.02)
Rexport	0.0153***	0.0191***	0.00283*	0.0150**	0.0219***	0.00325**
	(3.36)	(3.54)	(2.55)	(2.71)	(3.34)	(3.33)
RIPR	0.327***	0.322***	0.0525***	0.325***	0.345***	0.0348***
	(7.83)	(7.15)	(4.42)	(6.13)	(6.32)	(5.03)
ifdi	0.00240**	0.00291**	0.000282	0.00240**	0.00287**	0.000102
	(2.81)	(3.29)	(0.83)	(2.79)	(3.24)	(0.55)
gdp	0.00539***	0.00580***	0.000382	0.00537***	0.00584***	0.000106
	(6.28)	(6.61)	(1.64)	(6.26)	(6.64)	(0.46)
RD	-0.145***	-0.157***	-0.0129	-0.145***	-0.157***	-0.00660
	(-4.75)	(-5.10)	(-1.42)	(-4.73)	(-5.07)	(-1.11)
graduates	0.0812***	0.0766***	0.0155**	0.0813***	0.0759***	0.00951***
	(9.91)	(8.85)	(3.33)	(9.90)	(8.73)	(4.16)
Rexport_RIPR				0.000140	-0.00169	-0.000339
				(0.07)	(-0.74)	(-0.94)
Sargan test			133.69***			233.60***
AR(1)			0.020			0.029
AR(2)			0.111			0.091
_cons	3.863***	3.798***	0.917***	3.866***	3.771***	0.619***
	(24.71)	(37.58)	(7.70)	(24.50)	(35.06)	(4.42)
N	589	589	558	589	589	558

Note: This table reports the estimation results of the impact of export. We used OLS method and FE model to estimate Eqs (1) and (2) while we use system GMM one-step method to estimate Eqs (3) and (4). For system GMM method, we employ the lagged two-period international openness proxies as endogenous variables and other variables as predetermined variables. We report the chi2 statistics for Sargan test of over-identification and the P values for AR (1) and AR (2) test. It is assumed that the error term in system GMM model has significant first-order auto-correlation and insignificant second-order auto-autocorrelation. T statistics are reported in parentheses.

*, **, and *** indicates statistical significance at the 5%, 1%, and 0.1% level, respectively.

**Table 6:** Baseline results (*Rimport*).

	(1)	(2)	(3)	(4)	(5)	(6)
	OLS	FE	SGMM	OLS	FE	SGMM
L.INV1			0.848***			0.869***
			(24.23)			(34.92)
Rimport	0.0145***	0.0175***	0.00267**	0.0198***	0.0275***	0.00415***
	(3.71)	(3.61)	(3.06)	(3.81)	(4.01)	(4.07)
RIPR	0.279***	0.283***	0.0304*	0.337***	0.372***	0.0432***
	(6.27)	(6.16)	(2.74)	(5.77)	(5.91)	(5.09)
ifdi	0.00282***	0.00344***	0.000348	0.00248**	0.00309***	0.0000923
	(3.48)	(3.97)	(1.28)	(2.95)	(3.51)	(0.42)
gdp	0.00545***	0.00585***	0.000370	0.00538***	0.00581***	0.000266
	(6.35)	(6.66)	(1.65)	(6.27)	(6.64)	(1.32)
RD	-0.152***	-0.168***	-0.0136	-0.140***	-0.152***	-0.00704
	(-5.08)	(-5.50)	(-1.91)	(-4.55)	(-4.86)	(-1.05)
graduates	0.0816***	0.0777***	0.0143***	0.0806***	0.0748***	0.0125***
	(10.06)	(9.11)	(3.97)	(9.92)	(8.68)	(4.76)
Rimport_RIPR				-0.000981	-0.00144*	-0.000364**
				(-1.54)	(-2.06)	(-3.29)
Sargan test			95.72***			140.20***
AR(1)			0.027			0.027
AR(2)			0.116			0.115
_cons	3.906***	3.843***	0.873***	3.834***	3.703***	0.770***
	(26.72)	(41.68)	(5.22)	(24.62)	(32.37)	(5.72)
N	589	589	558	589	589	558

Note: This table reports the estimation results of the impact of imports. We used OLS method and FE model to estimate Eqs (1) and (2) while we use system GMM one-step method to estimate Eqs (3) and (4). For system GMM method, we employ the lagged two-period international openness proxies as endogenous variables and other variables as predetermined variables. We report the chi2 statistics for Sargan test of over-identification and the P values for AR (1) and AR (2) test. It is assumed that the error term in system GMM model has significant first-order auto-correlation and insignificant second-order auto-autocorrelation. T statistics are reported in parentheses.

*, **, and *** indicates statistical significance at the 5%, 1%, and 0.1% level, respectively.

According to the results in column (3) in Tables 5 and 6, a 1-unit increase in international openness measured by export and import can significantly promote regional innovation by 0.283% and 0.267%, respectively. The coefficients of the IPR variable are still consistent with previous conclusions even after the international openness variables are replaced with export and import, indicating that stronger IPR protection can stimulate more innovation activities. As for the interaction term, only import and IPR protection have a mediation effect between each other as its GMM estimator is negative and significant in column (6) in Table 5, indicating that stronger IPR protection could intensify the negative effect of import competition to hinder innovative domestic activities.

**Fig 1:**
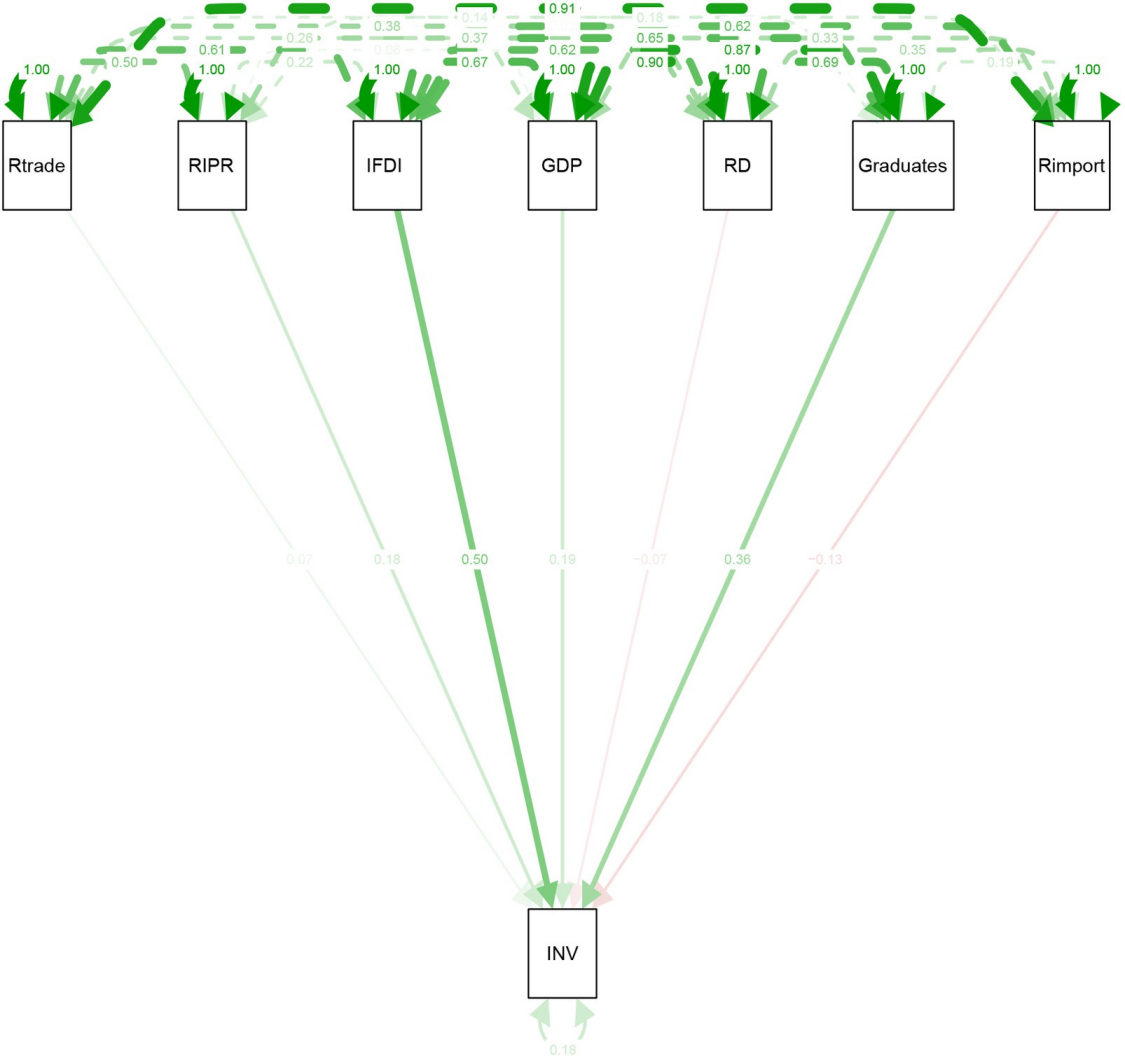
Causal relationship of all the variables depicted by path analysis. Source: Authors’ drawn.

The results of path analysis corroborate the above interpretations. The significant positive impact of graduates, GDP, *ifdi*, and *RIPR* has been confirmed by path analysis. The pictorial illustration of the path model is given in Fig 1.

### 4.2. Empirical results by regions

The pooled dataset of all regions may bring heteroscedasticity to the estimated models because there are huge gaps in innovating activities, economic development, IPR protection and R&D funding for different regions in China. Therefore, we estimated Eq (2) for three subsamples and the results are displayed in Tables 7–9.

**Table 7:** System GMM estimates by region (*Rtrade*).

	(1)	(2)	(3)	(4)	(5)	(6)
	Eastern Provinces	Central Provinces	Western Provinces	Eastern Provinces	Central Provinces	Western Provinces
L.INV1	0.852***	0.808***	0.802***	0.868***	0.826***	0.930***
	(24.33)	(32.36)	(14.82)	(46.38)	(33.47)	(22.19)
Rtrade	0.00170**	0.00699	-0.00102	0.00210*	-0.00998	0.00168
	(3.76)	(1.51)	(-0.17)	(3.13)	(-0.74)	(0.22)
RIPR	0.0265	0.0773	0.0386	0.0376*	-0.244	0.151
	(1.88)	(0.85)	(0.93)	(2.58)	(-0.93)	(1.58)
Rtrade_RIPR				-0.000156	0.0313	-0.0121
				(-1.51)	(1.29)	(-1.42)
Control variables	Yes	Yes	Yes	Yes	Yes	Yes
Sargan test	99.93***	99.34***	93.68***	137.34***	119.97***	127.18***
AR(1)	0.069	0.009	0.000	0.062	0.010	0.000
AR(2)	0.002	0.011	0.873	0.002	0.010	0.923
_cons	0.918**	1.066***	0.919***	0.825***	1.161***	0.450**
	(4.52)	(8.59)	(5.13)	(6.44)	(5.93)	(2.89)
*N*	198	144	216	198	144	216

Note: This table reports the estimation results of the impact of overall trade by regions. We used only system GMM one-step method for estimation. For system GMM method, we employ the lagged two-period international openness proxies as endogenous variables and other variables as predetermined variables. We report the chi2 statistics for Sargan test of over-identification and the P values for AR (1) and AR (2) test. It is assumed that the error term in system GMM model has significant first-order auto-correlation and insignificant second-order auto-autocorrelation. T statistics are reported in parentheses.

*, **, and *** indicates statistical significance at the 5%, 1%, and 0.1% level, respectively.

**Table 8:** System GMM estimates by region (*Rexport*).

	(1)	(2)	(3)	(4)	(5)	(6)
	Eastern Provinces	Central Provinces	Western Provinces	Eastern Provinces	Central Provinces	Western Provinces
L.INV1	0.839***	0.836***	0.831***	0.857***	0.860***	0.906***
	(29.12)	(23.36)	(16.19)	(34.22)	(25.67)	(24.08)
Rexport	0.00356**	-0.00871	-0.00200	0.00343**	-0.0373	0.00306
	(3.29)	(-1.19)	(-0.32)	(3.22)	(-1.78)	(0.40)
RIPR	0.0429*	0.0340	0.0292	0.0368**	-0.380	0.0821
	(3.14)	(0.40)	(0.69)	(3.84)	(-1.40)	(1.19)
Rexport_RIPR				0.0000741	0.0724	-0.00940
				(0.28)	(1.67)	(-1.14)
Control variables	Yes	Yes	Yes	Yes	Yes	Yes
Sargan test	105.75***	94.68***	107.22***	126.80***	113.28***	143.60***
AR(1)	0.056	0.010	0.000	0.049	0.011	0.000
AR(2)	0.002	0.010	0.882	0.002	0.010	0.937
_cons	0.967***	1.048***	0.829***	0.885***	1.122***	0.537***
	(6.66)	(5.69)	(4.74)	(9.56)	(4.65)	(3.85)
*N*	198	144	216	198	144	216

Note: This table reports the estimation results of the impact of export by regions. We used only system GMM one-step method for estimation. For system GMM method, we employ the lagged two-period international openness proxies as endogenous variables and other variables as predetermined variables. We report the chi2 statistics for Sargan test of over-identification and the P values for AR (1) and AR (2) test. It is assumed that the error term in system GMM model has significant first-order auto-correlation and insignificant second-order auto-autocorrelation. T statistics are reported in parentheses.

*, **, and *** indicates statistical significance at the 5%, 1%, and 0.1% level, respectively.

**Table 9:** System GMM estimates by region (*Rimport*).

	(1)	(2)	(3)	(4)	(5)	(6)
	Eastern Provinces	Central Provinces	Western Provinces	Eastern Provinces	Central Provinces	Western Provinces
L.INV1	0.859***	0.792***	0.795***	0.852***	0.764***	0.896***
	(17.76)	(17.93)	(14.65)	(39.83)	(21.03)	(20.27)
Rimport	0.00250*	0.0296	-0.00651	0.00329*	0.00454	0.0260
	(2.62)	(1.79)	(-0.31)	(2.54)	(0.30)	(0.90)
RIPR	0.0205	0.0758	0.0330	0.0433**	-0.103	0.177
	(1.25)	(0.98)	(0.78)	(3.21)	(-0.52)	(1.78)
Rimport_RIPR				-0.000266	0.0448	-0.0434
				(-1.78)	(1.20)	(-1.69)
Control variables	Yes	Yes	Yes	Yes	Yes	Yes
Sargan test	90.16***	89.06***	68.84***	85.54***	117.62***	106.14***
AR(1)	0.082	0.011	0.000	0.000	0.010	0.000
AR(2)	0.002	0.014	0.878	0.001	0.012	0.957
_cons	0.909**	1.085***	0.957***	0.917***	1.308***	0.510**
	(3.20)	(5.69)	(5.16)	(5.37)	(7.74)	(3.00)
*N*	198	144	216	198	144	216

Note: This table reports the estimation results of the impact of import by regions. We used only system GMM one-step method for estimation. For system GMM method, we employ the lagged two-period international openness proxies as endogenous variables and other variables as predetermined variables. We report the chi2 statistics for Sargan test of over-identification and the P values for AR (1) and AR (2) test. It is assumed that the error term in system GMM model has significant first-order auto-correlation and insignificant second-order auto-autocorrelation. T statistics are reported in parentheses.

*, **, and *** indicates statistical significance at the 5%, 1%, and 0.1% level, respectively.

As can be seen in Table 7, overall trade openness only has a significant impact on the number of invention patent grants of eastern provinces but not central provinces and western provinces. The effects of IPR protection also show the same pattern when interaction term is added into the model. With trade accounting for a large proportion to local economy, eastern provinces can benefit more from the international market and IPR protection enhancement. As shown in Tables 8 and 9, the impacts of export and import present the same patterns to trade openness, which concludes that the innovating activities can be significantly advanced only for eastern provinces but not for central and western provinces by export and import. The direction of the effects of export and import is also consistent with the baseline results, while it also implies the positive effect of export and import on innovation are majorly manifested in more developed areas like eastern provinces.

## 5. Robustness checks

To check the robustness of our empirical findings, we are going to set international openness, regional IPR protection and GDP as endogenous variables in the GMM estimation. The estimation results are displayed in Table 10. It can be seen that the GMM estimators for international openness proxies are still positive and significant after considering the potential endogeneity problem of international openness, regional IPR protection and GDP. Therefore, our empirical findings show strong consistency across different model specifications.

**Table 10:** Robustness check.

	(1)	(2)	(3)
	INV	INV	INV
L.INV1	0.876***	0.887***	0.865***
	(50.35)	(46.12)	(36.33)
Rtrade	0.00135**		
	(3.41)		
Rexport		0.00272*	
		(2.61)	
Rimport			0.00250**
			(2.96)
RIPR	0.0356**	0.0400***	0.0234
	(3.46)	(4.09)	(1.70)
Control variables	Yes	Yes	Yes
Sargan test	277.02***	274.59***	281.41***
AR(1)	0.026	0.027	0.029
AR(2)	0.106	0.100	0.108
_cons	0.750***	0.699***	0.809***
	(7.01)	(5.76)	(6.13)
*N*	558	558	558

Note: This table reports the estimation results of robustness checks. W We used only system GMM one-step method for estimation. For system GMM method, we employ the lagged one-period international openness and IPR protection proxies and GDP variable as endogenous variables and other variables as predetermined variables. We report the chi2 statistics for Sargan test of over-identification and the P values for AR (1) and AR (2) test. It is assumed that the error term in system GMM model has significant first-order auto-correlation and insignificant second-order auto-autocorrelation. T statistics are reported in parentheses.

*, **, and *** indicates statistical significance at the 5%, 1%, and 0.1% level, respectively.

## 6. Conclusions

Overall trade and a higher share of exports and imports to GDP have significant and robust positive effects across multiple model specifications, demonstrating that greater international openness can encourage regional innovative activities in China. The results of the system GMM model match the causal relationship of all the variables depicted by path analysis. Increased intellectual property protection allows each region to reap the economic benefits of innovation and spend more in R&D. A large portion of local innovating activities can also be explained by the lag effect of regional innovation capability. The positive effect of trade openness on innovation is most pronounced in developed areas, such as eastern provinces, according to our subsample regressions. Increased export opportunities and import competition tend to motivate Chinese provinces to invest more in innovating activities and develop their own technology by "learning by exporting" instead of just importing foreign technology.

Considering the huge gap in each province’s conditions, empirical results in subsamples show that overall trade openness and IPR protection can only promote the innovation of eastern provinces but not central provinces and western provinces. The impacts of export and import present the same patterns to trade openness, which concludes that the innovating activities can be significantly advanced only for eastern provinces but not for central and western provinces by export and import. It also implies the positive effect of export and import on innovation are majorly manifested in developed areas like eastern provinces.

Finally, to improve regional innovation capacity, the local government should implement more open economic policies and stronger IPR protection according to their specific conditions. For regions with better economic performances, promoting international trade and IPR protection will be conducive to improving local innovation ability. Accordingly, regions with poorer economies should focus on increasing local R&D investments and attracting more talents.

## Supporting information

S1 Dataset (XLS)
